# Supplementary material for: Asymbiotic mass production of the arbuscular mycorrhizal fungus Rhizophagus clarus
Source: Commun Biol. 2022 Jan 12;5:43. doi: 10.1038/s42003-021-02967-5 (PMC8755765; doi:10.1038/s42003-021-02967-5)
Supplement: Supplementary file 2 — Supplementary Information [file 42003_2021_2967_MOESM2_ESM.pdf]

Supplementary Information for "Asymbiotic mass production of the arbuscular mycorrhizal fungus *Rhizophagus clarus* HR1"

Sachiko Tanaka, Kayo Hashimoto, Yuuki Kobayashi, Koji Yano, Taro Maeda, Hiromu Kameoka, Tatsuhiro Ezawa, Katsuharu Saito, Kohki Akiyama, Masayoshi Kawaguchi*

*Corresponding author:

E-mail address: masayosi@nibb.ac.jp

This Supplementary Information file contains:

Supplementary Figure 1–10

Supplementary Table 1, 2

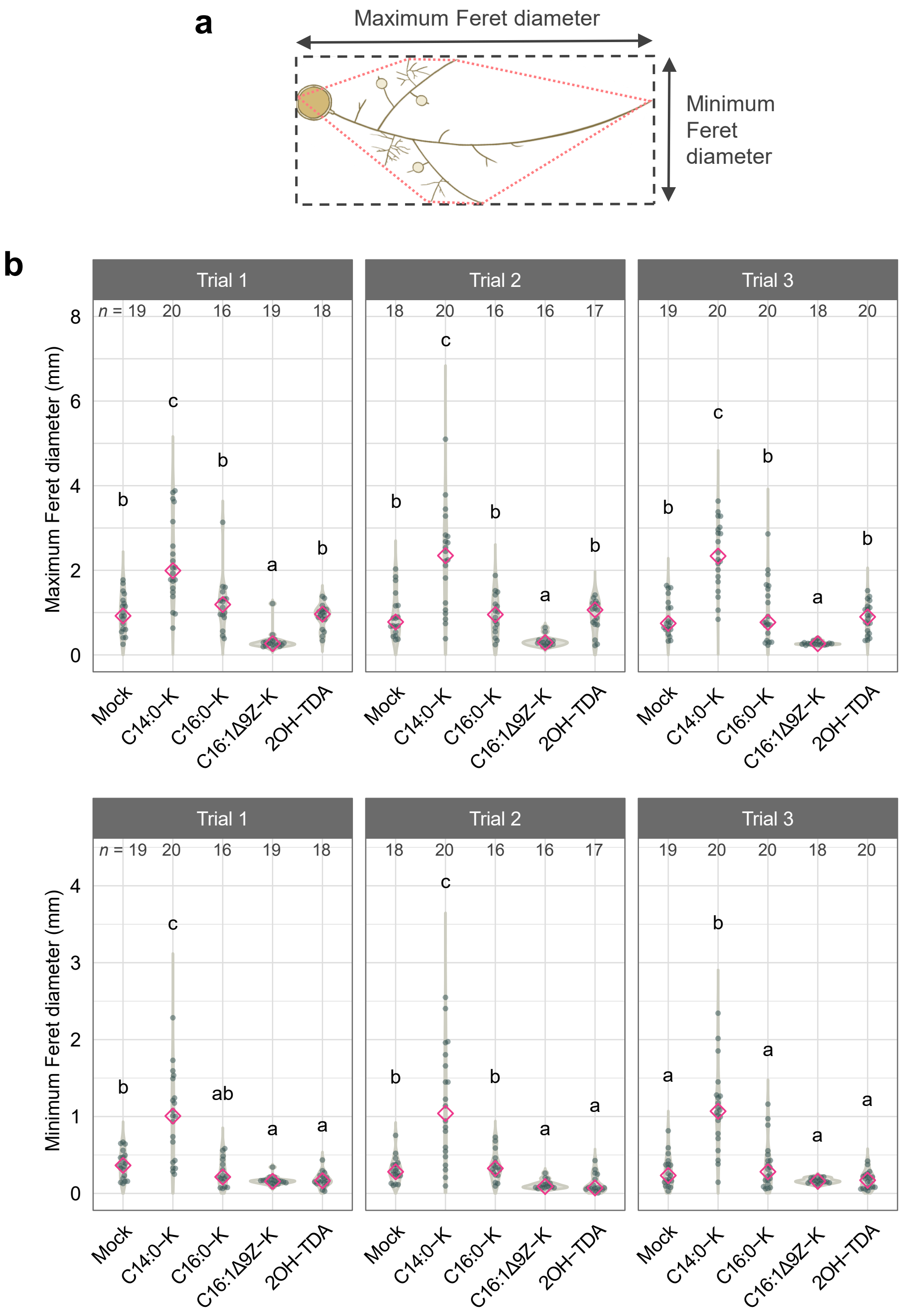

Supplementary Figure 1. Effects of fatty acids on asymbiotic cultures of *R. clarus*. **a**, The outline of the measuring method using the maximum and minimum Feret diameters (explained in dashed box) for fungal spread hyphae. Feret diameters were analysed using ImageJ software from a polygon drawn by connecting the tips of hyphae with straight lines (shown in dotted red line). **b**, Maximum and minimum Feret diameter of *R. clarus* in conditions supplemented with 100 μM fatty acid at 6 weeks after incubation (WAI). Diamonds indicate medians. Statistical significance was calculated using the Wilcoxon rank-sum test with Bonferroni correction. *Different letters* indicate significant differences ($p < 0.05$). *p*-values are described in Supplementary Data 2. C14:0-K, potassium myristate. C16:0-K, potassium palmitate. C16:1Δ9Z-K, potassium palmitoleate. 2OH-TDA, 2-hydroxytetradecanoic acid.

a

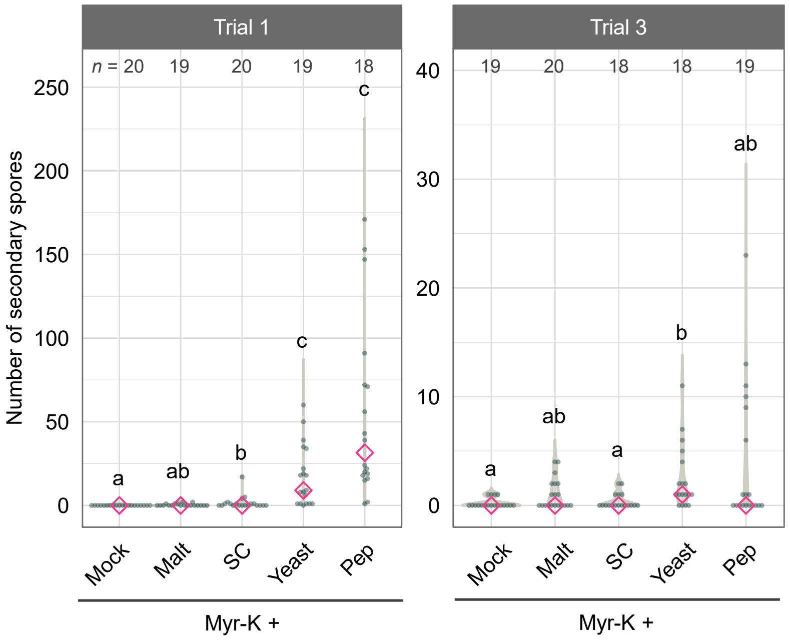
Trial 1
Trial 3
n = 20
19
20
19
18
19
20
18
18
19
Number of secondary spores
250
200
150
100
50
0
40
30
20
10
0
a
ab
b
c
c
a
ab
a
b
ab
Mock
Malt
SC
Yeast
Pep
Mock
Malt
SC
Yeast
Pep
Myr-K +
Myr-K +

b

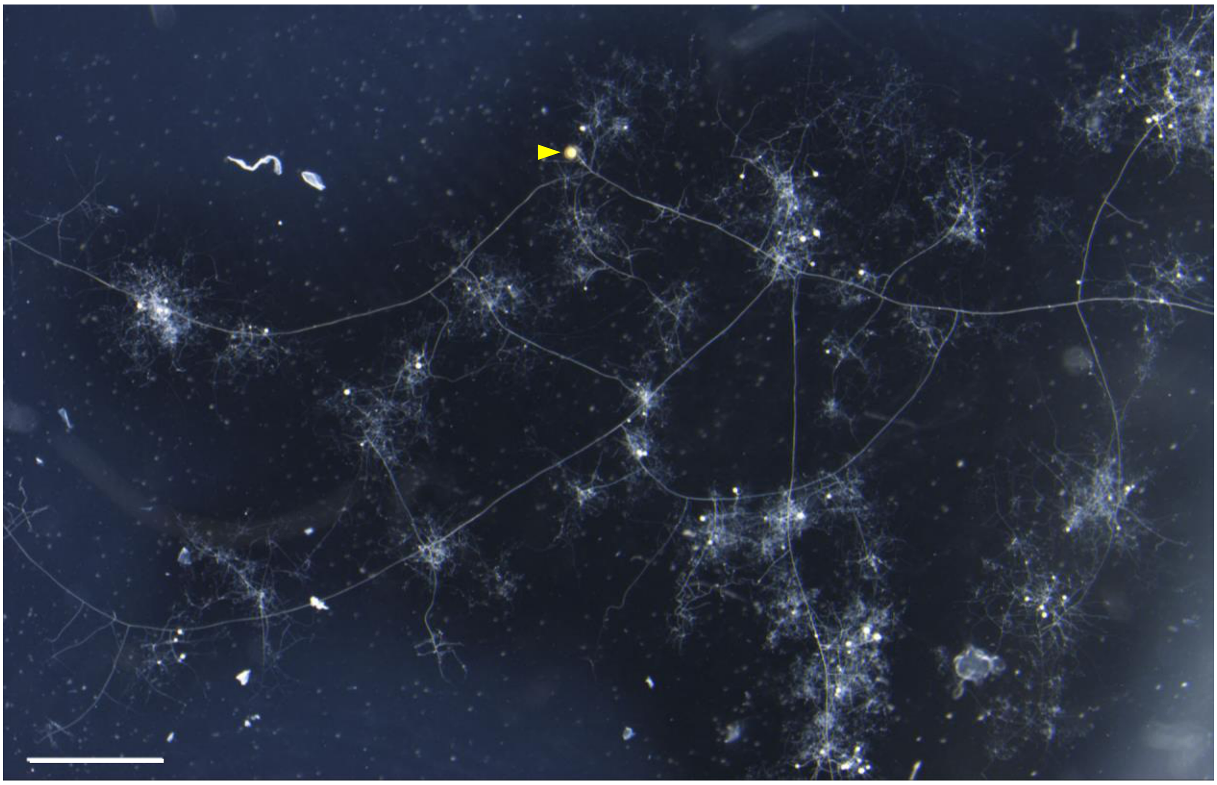

c

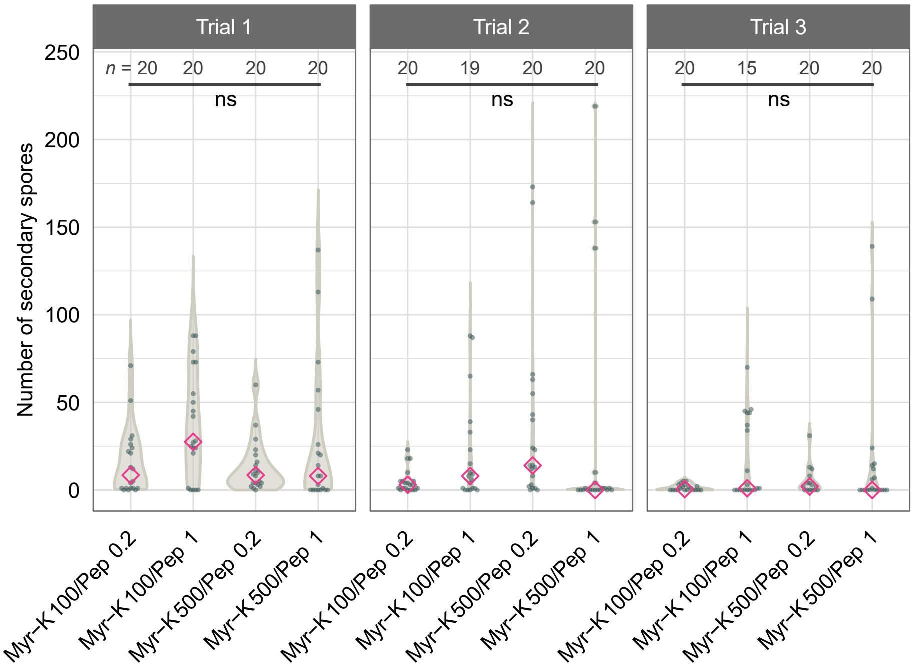

Supplementary Figure 2. Effects of organic nitrogen on asymbiotic cultures of *R. clarus*. a, Another two trials of Fig. 1d (trial 2); numbers of secondary spores in medium supplemented with 0.2 g l^{-1} organic nitrogen in the presence of Myr-K at 6 WAI. Diamonds indicate medians. Statistical significance was calculated using the Wilcoxon rank-sum test with Bonferroni correction. *Different letters* indicate significant differences ($p < 0.05$). *p*-values are described in Supplementary Data 2. Malt, malt extract. Pep, peptone. SC, SC dropout. Yeast, yeast extract. **b**, Low magnification image of *R. clarus* cultured in the medium containing Myr-K and peptone in Fig. 1a. An arrowhead is a parent spore. Bar = 2 mm. **c**, Comparison of secondary spore number among different combinations of Myr-K (100 or 500 μM) and peptone (0.2 or 1.0 g l^{-1}) at 6 WAI. Diamonds indicate medians. Statistical significance was calculated using the Wilcoxon rank-sum test with Bonferroni correction ($p < 0.05$). ns, not significant. *p*-values are described in Supplementary Data 2.

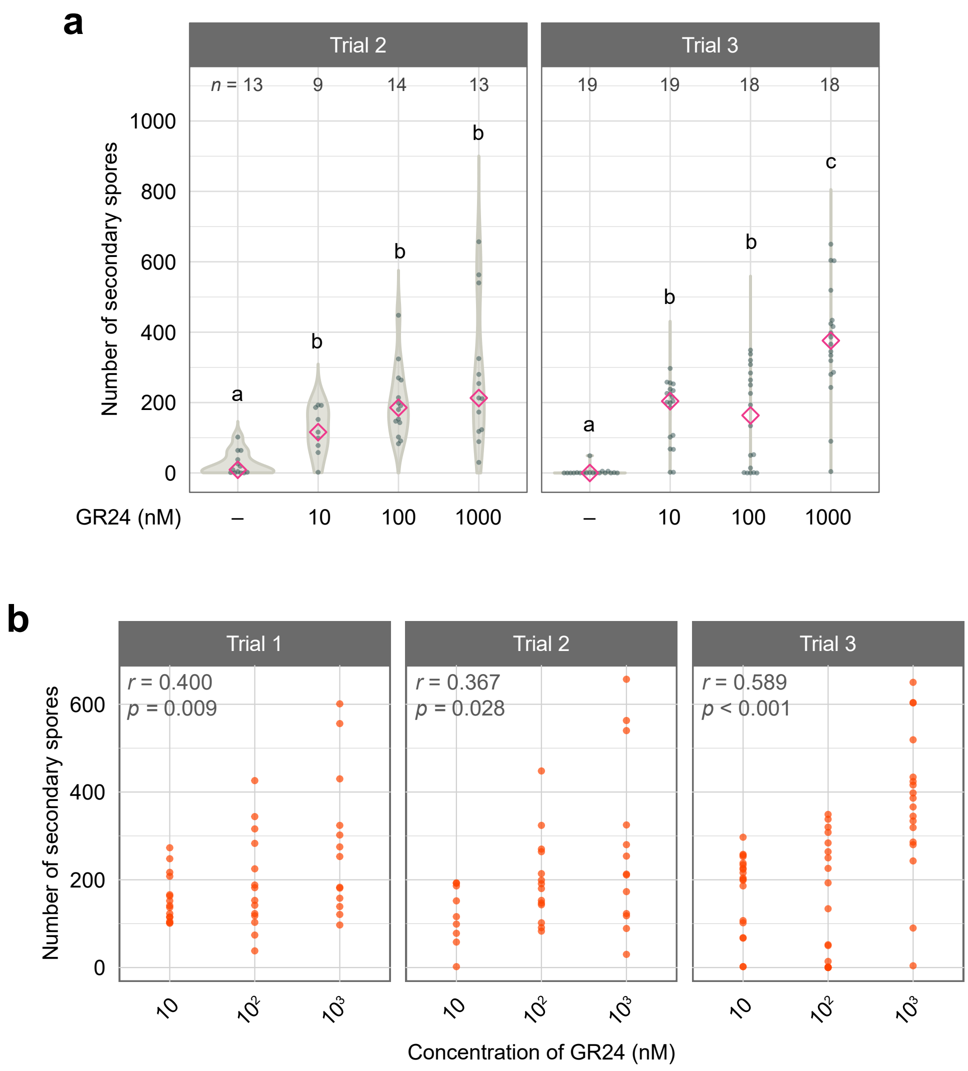

Supplementary Figure 3. Effects of GR24 on asymbiotic cultures of *R. clarus*. a, Another two trials of Fig. 2b (trial 1); numbers of secondary spores in cultures with different concentrations of GR24 (10, 100, 1000 nM) in the presence of 500 µM Myr-K and 1 g l^{-1} peptone at 6 WAI. Diamonds indicate medians. Statistical significance was calculated using the Wilcoxon rank-sum test with Bonferroni correction. *Different letters* indicate significant differences ($p < 0.05$). *p*-values are described in Supplementary Data 2. **b**, Correlation between GR24 concentrations and the number of secondary spores. *r* values are Pearson's correlation coefficient.

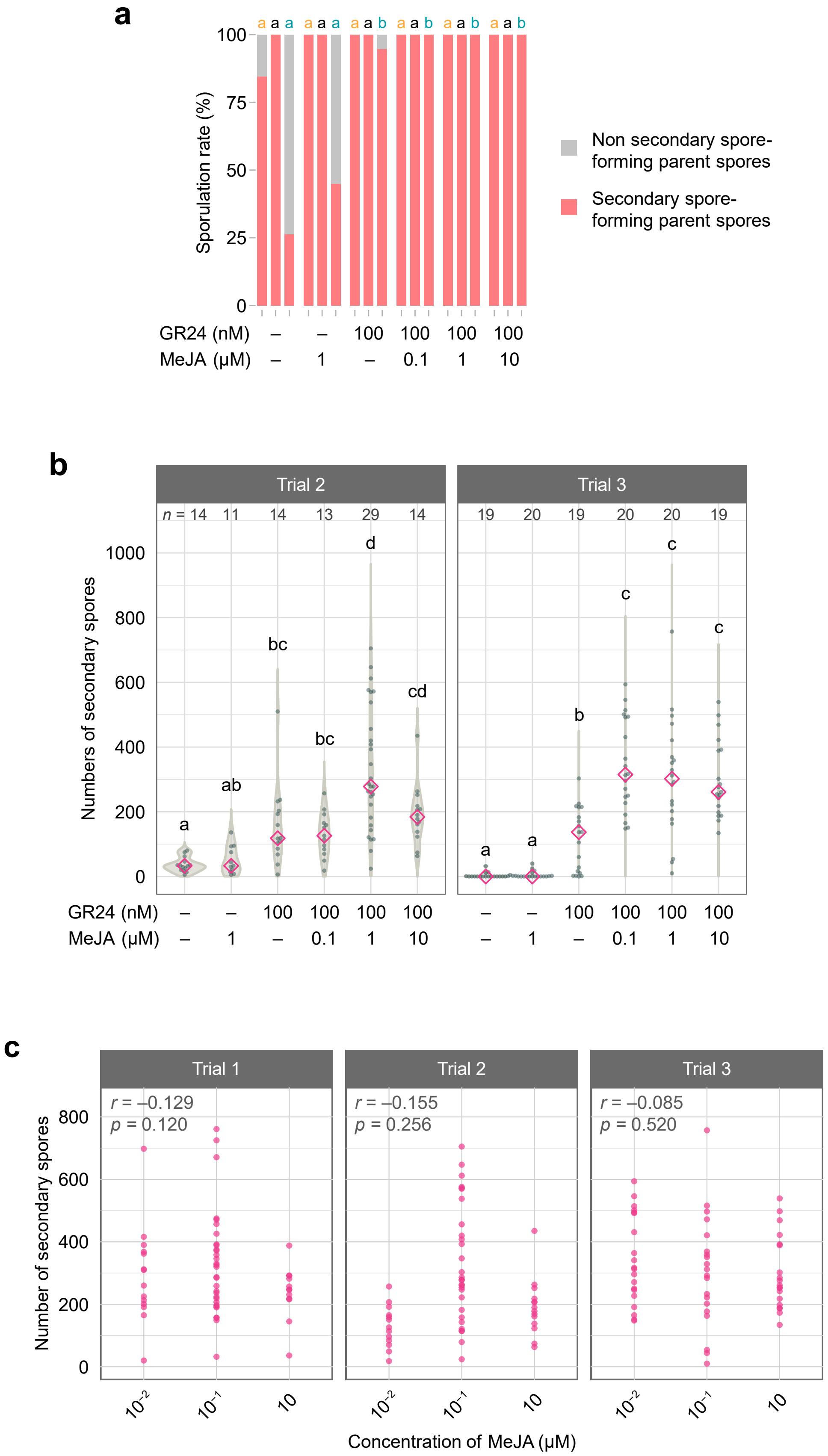
a
Sporulation rate (%)
100
75
50
25
0
a a a a a a a a b a a b a a b a a b
GR24 (nM) – – 100 100 100 100
MeJA (μM) – 1 – 0.1 1 10
Non secondary spore-forming parent spores
Secondary spore-forming parent spores
b
Trial 2
Trial 3
n = 14 11 14 13 29 14
19 20 19 20 20 19
Numbers of secondary spores
1000
800
600
400
200
0
a ab bc bc d cd
a a b c c c
GR24 (nM) – – 100 100 100 100 – – 100 100 100 100
MeJA (μM) – 1 – 0.1 1 10 – 1 – 0.1 1 10
c
Trial 1
Trial 2
Trial 3
r = –0.129
p = 0.120
r = –0.155
p = 0.256
r = –0.085
p = 0.520
Number of secondary spores
800
600
400
200
0
10⁻² 10⁻¹ 10
Concentration of MeJA (μM)

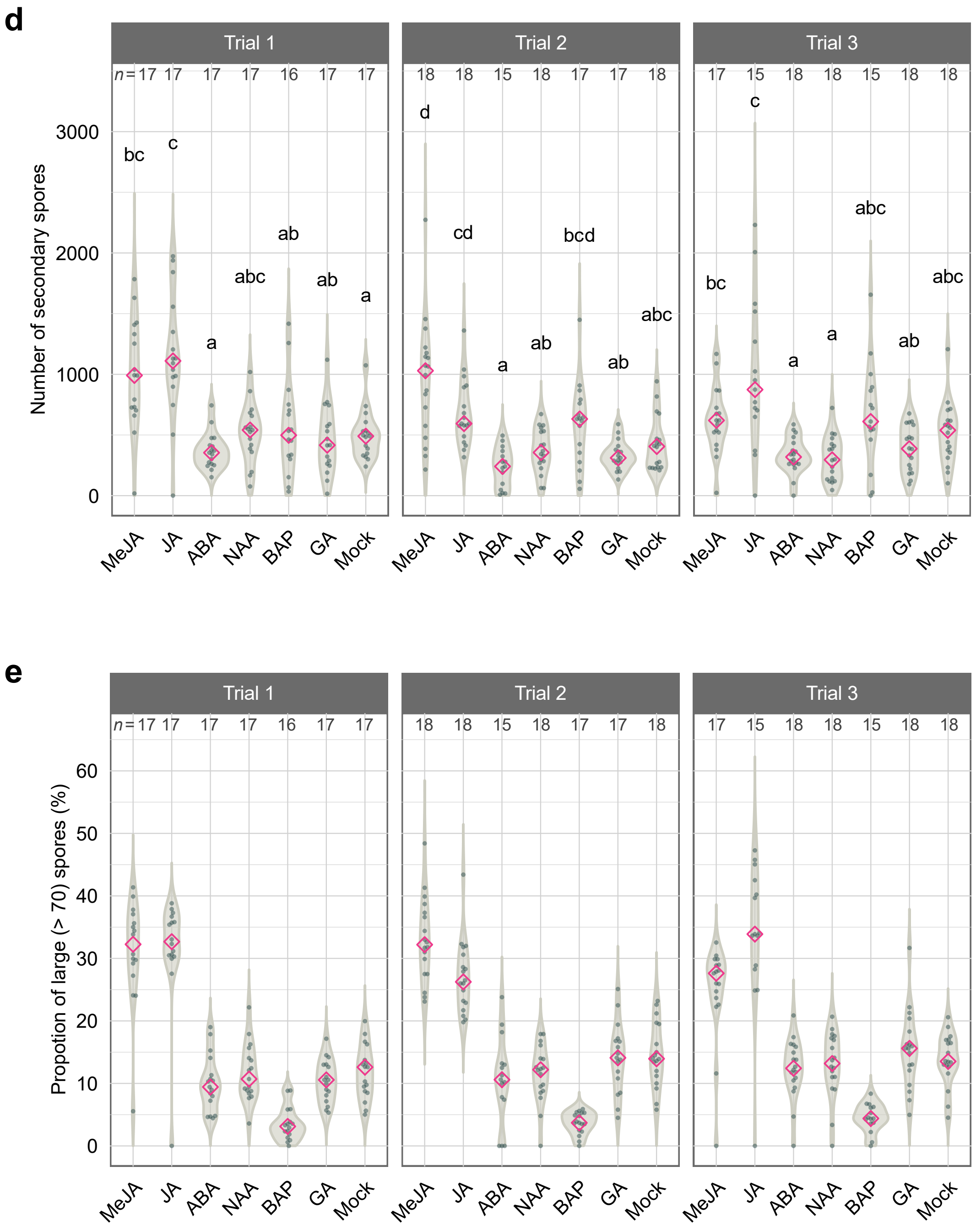
d
Trial 1
Trial 2
Trial 3
n = 17 17 17 17 16 17 17
18 18 15 18 17 17 18
17 15 18 18 15 18 18
Number of secondary spores
0
1000
2000
3000
bc c a abc ab ab a
d cd a ab bcd ab abc
bc c a a abc ab abc
MeJA JA ABA NAA BAP GA Mock
e
Trial 1
Trial 2
Trial 3
n = 17 17 17 17 16 17 17
18 18 15 18 17 17 18
17 15 18 18 15 18 18
Propotion of large (> 70) spores (%)
0
10
20
30
40
50
60
MeJA JA ABA NAA BAP GA Mock

f

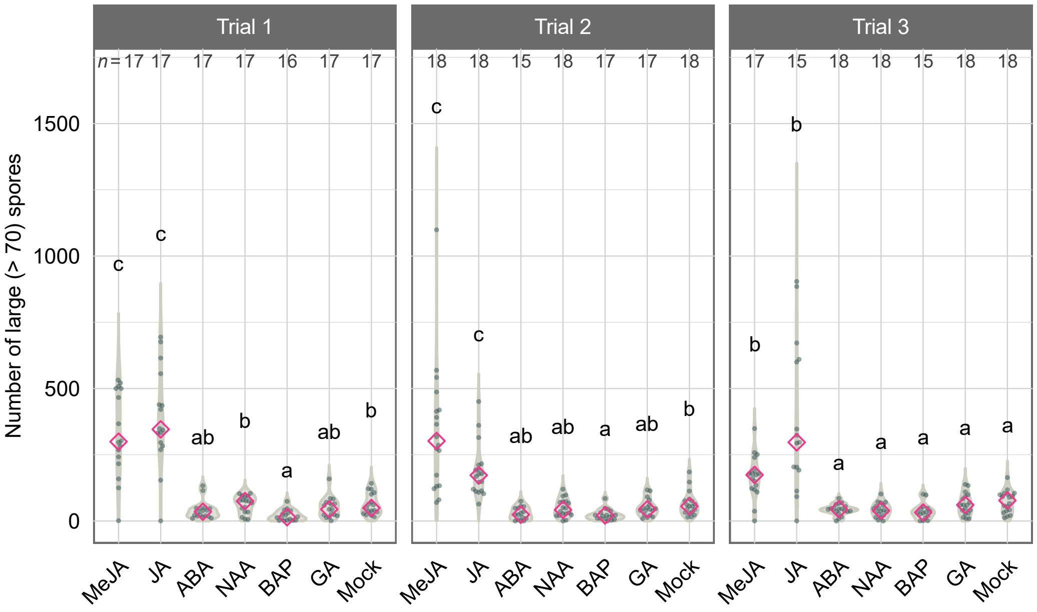

Supplementary Figure 4. Effects of GR24 and methyl jasmonate on asymbiotic cultures of *R. clarus*. **a**, Sporulation rates, percentage of secondary spore-forming parent spores relative to germinated patent spores, in medium containing 100 nM GR24 or MeJA (0.1, 1, 10 µM) or both at 6 WAI. Three bars in each condition are trial 1 to 3, respectively from the left. *Different letters* above of the graph indicate significant differences among treatments in each trial using Fisher's exact test with Bonferroni correction ($p < 0.05$). **b**, Another two trials of Fig. 3a (trial 1); asymbiotic culture experiments with GR24 and MeJA as before. Diamondsindicate medians. Statistical significance was calculated using the Wilcoxon rank-sum test with Bonferroni correction. *Different letters* indicate significant differences ($p < 0.05$). *p*-values are described in Supplementary Data 2. **c**, Correlation between MeJA concentrations and the number of secondary spores. *r* values are Pearson's correlation coefficient. **d–f**, Effects of six phytohormones on *R. clarus* asymbiotic culture at 8 WAI. Numbers of secondary spores in cultures with different phytohormones (1 µM each) in the presence of 500 µM Myr-K, 1 g l^{-1} peptone and 100 nM GR24. ABA, abscisic acid. BAP, 6-benzylaminopurine. GA, gibberellin A_3. JA, jasmonic acid. NAA, 1-napthoxyl acetic acid. Spores (> 30 µm in diameter) per spore were automatically counted (**d**) (see Materials and Methods). Diamonds indicate medians. Statistical significance was calculated using the Wilcoxon rank-sum test with Bonferroni correction. *Different letters* indicate significant differences ($p < 0.05$). *p*-values are described in Supplementary Data 2. The percentage of large (>70 µm in diameter) spores (**e**) and the number of large spores (**f**) in cultures with different phytohormones (1 µM each) in the presence of 100 nM GR24. Diamonds indicate medians. Statistical significance was calculated using the Wilcoxon rank-sum test with Bonferroni correction. *Different letters* indicate significant differences ($p < 0.05$). *p*-values are described in Supplementary Data 2.

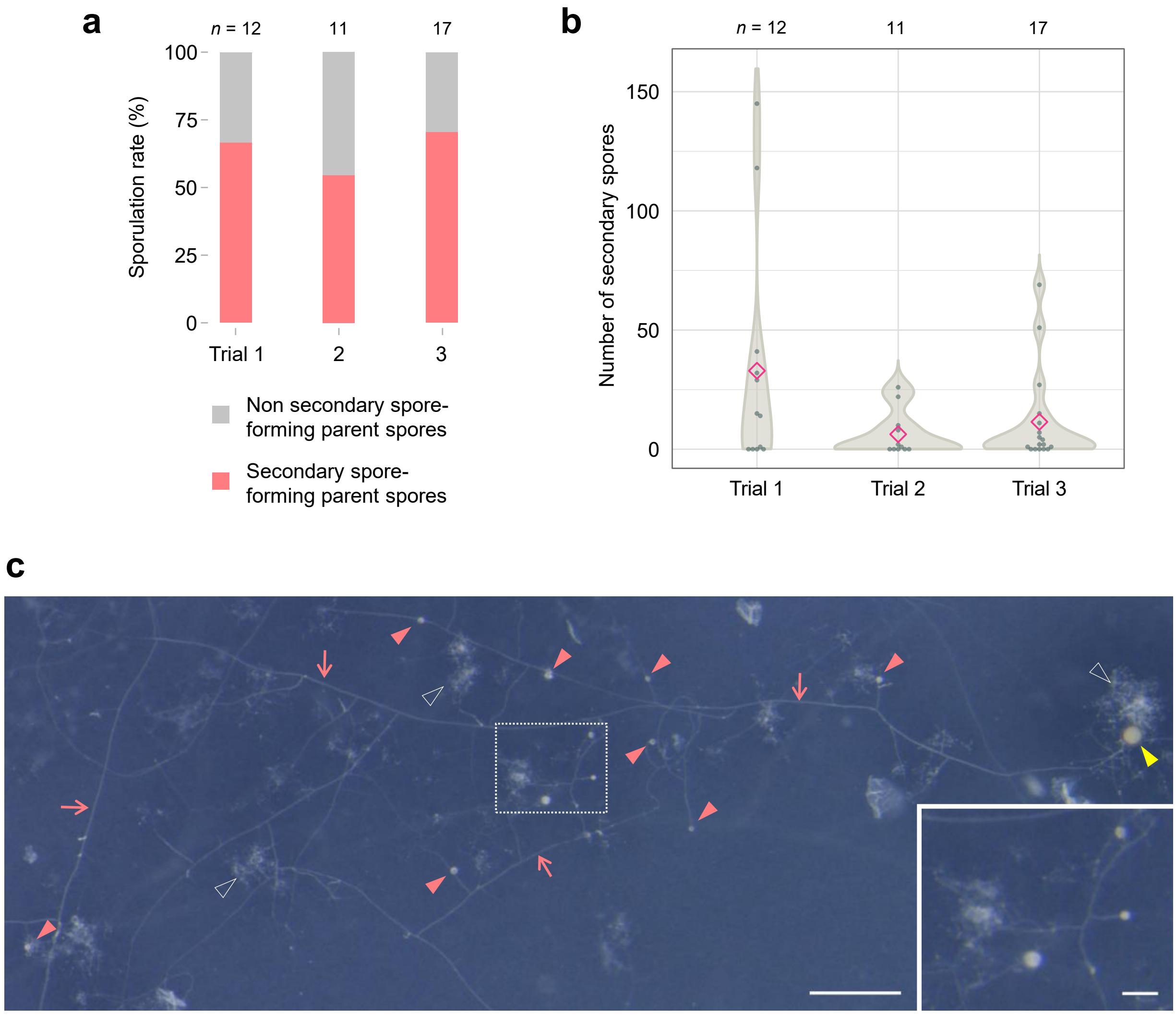

Supplementary Figure 5. Asymbiotic culture of *R. irregularis* DAOM197198. a, Sporulation rates, percentage of secondary spore-forming parent spores relative to germinated patent spores, in TGM medium at 8 WAI. **b**, Numbers of secondary spores produced in TGM medium at 8 WAI. Diamonds indicate means. **c**, *R. irregularis* secondary spores generated on TGM medium at 8 WAI. The inset is the magnified image of the dotted box. Yellow and red arrowheads indicate parent and newly generated secondary spores, respectively. Arrows indicate runner hyphae. Outlined arrowheads indicate small densely packed coil structures. Bars indicate 500 (large image) and 100 µm (inset), respectively.

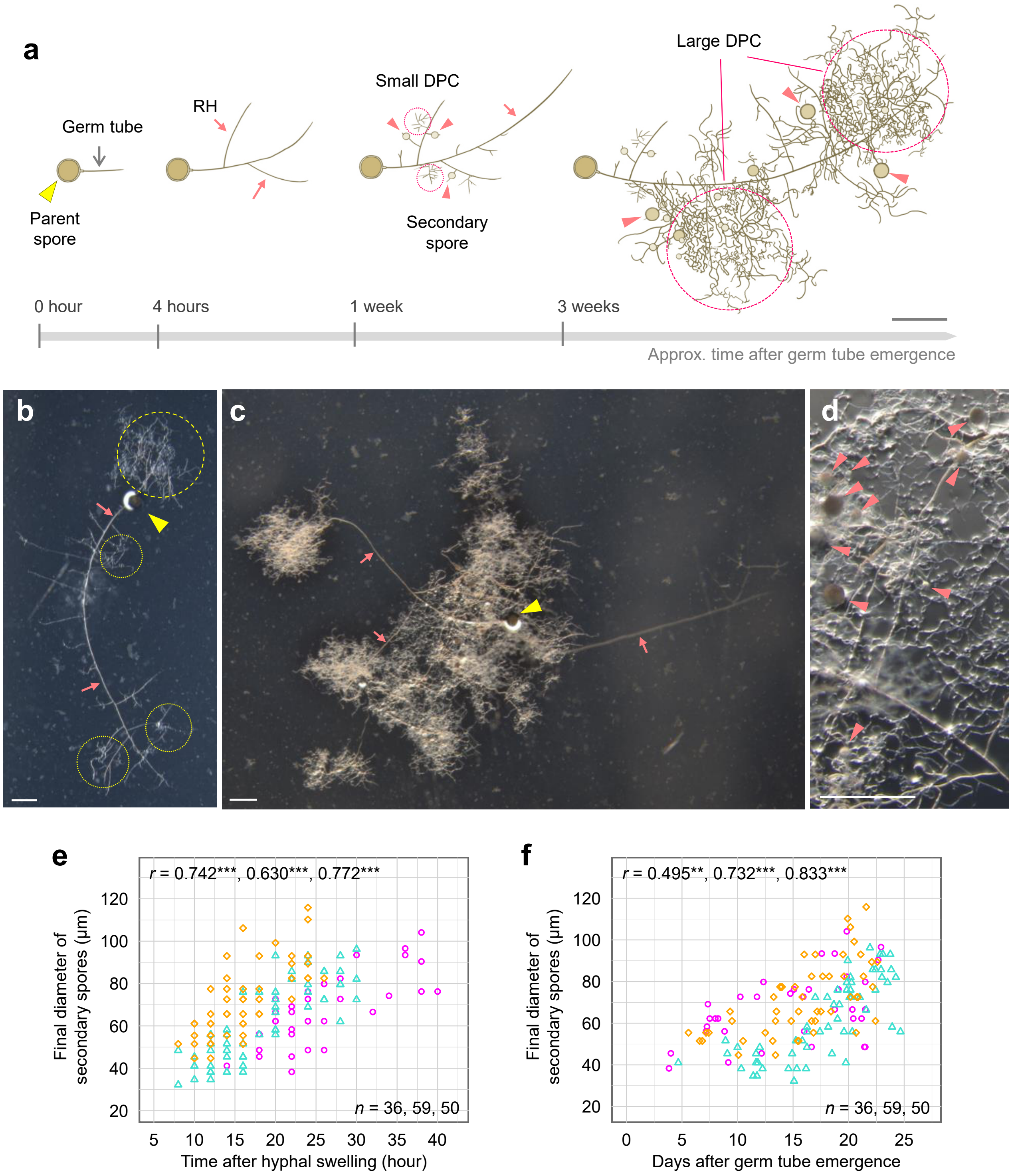

Supplementary Figure 6. Time-lapse analysis of *R. clarus* growth under asymbiotic conditions. a–d, A schematic diagram (**a**) and representative images (**b–d**) showing *R. clarus* growth in TGM medium. Yellow and pink arrowheads indicate parent and secondary spores, respectively. Grey and pink arrows indicate germ tubes and runner hyphae (RH), respectively. Dotted and dashed circles indicate small and large DPC (densely packed coils), respectively. Bars = 500 µm. **b**, Small DPCs and a developing large DPC at 11 days after germ tube emergence. **c**, Developed large DPCs. **d**, Magnified image of a large DPC. **e–f.** Development of secondary spores in TGM medium. Only focused images were analysed. Circles, triangles and diamonds indicate trials 1 to 3, respectively. *r* values are Pearson's correlation coefficient. The three values are trials 1 to 3, respectively from the left. ***, $p < 0.001$; **, $0.001 \leq p < 0.01$. *p*-values are described in Supplementary Data 2. **e**, Correlation between the final diameter of secondary spores and time required for the spore development after hyphal swelling. **f**, Correlation between the final diameter of secondary spores and days after germ tube emergence.

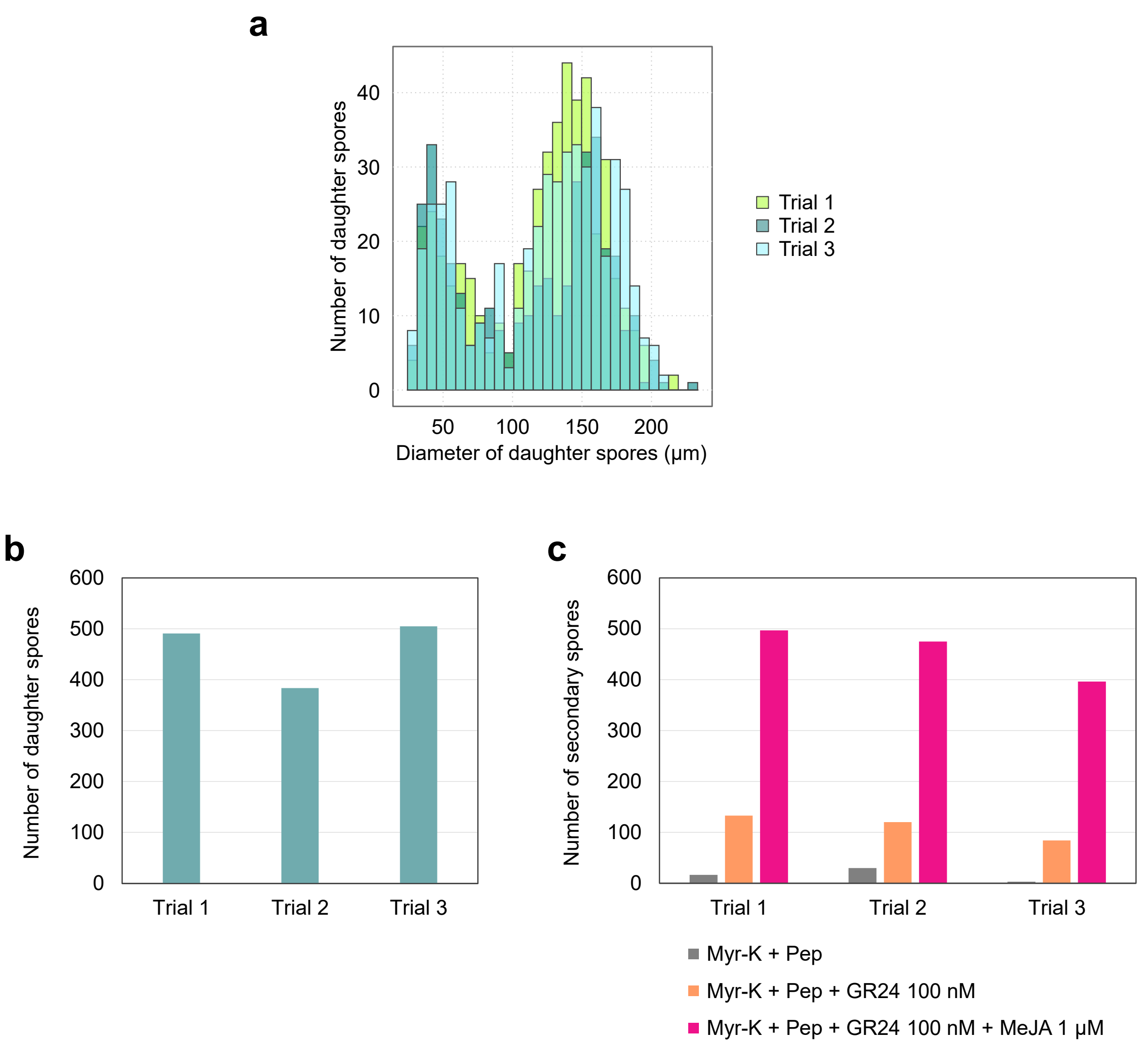

Supplementary Figure 7. Comparison of spores generated by asymbiotic and *in vitro* monoxenic culture. a, Size distribution of daughter spores produced on the extraradical hyphae emerged from carrot hairy roots inoculated with a single spore. **b**, Numbers of daughter spores generated from a single spore by *in vitro* monoxenic culture at 8 WAI. **c**, Numbers of secondary spores (> 30 μm in diameter) per a parent spore at 8 WAI. The total number of secondary spores produced from 8 parent spores was automatically counted using Ilastik and ImageJ software (see Materials and Methods). The values were that the total number divided by the number of parent spores.

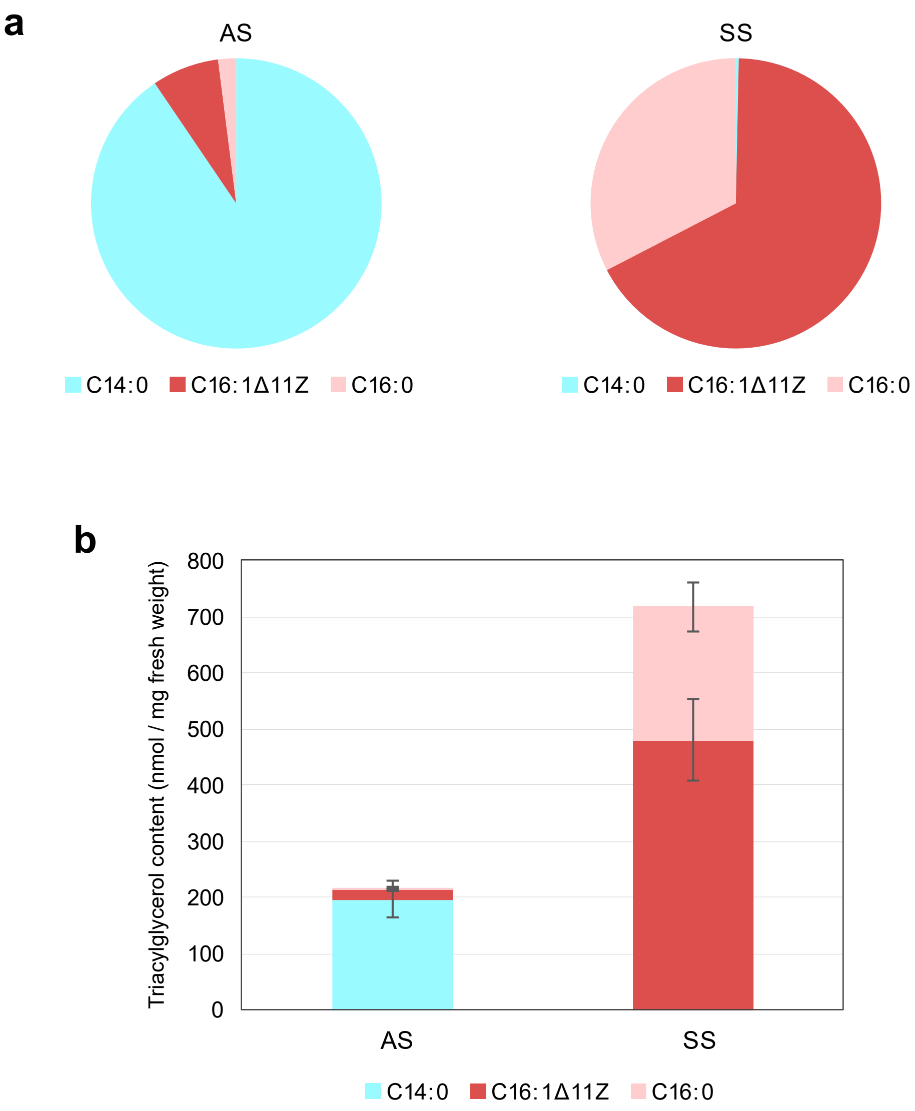

Supplementary Figure 8. Comparison of the fatty acid composition of AM fungal triacylglycerol between asymbiotically-generated spores and symbiotically-generated spores. Composition of three fatty acids (**a**) and fresh weight of triacylglycerol (**b**) in each spore. Error bars show the standard error. AS, asymbiotically-generated spores. SS, symbiotically-generated spores. C14:0, myristic acid. C16:0, palmitic acid. C16:1Δ11Z, palmitvaccenic acid.

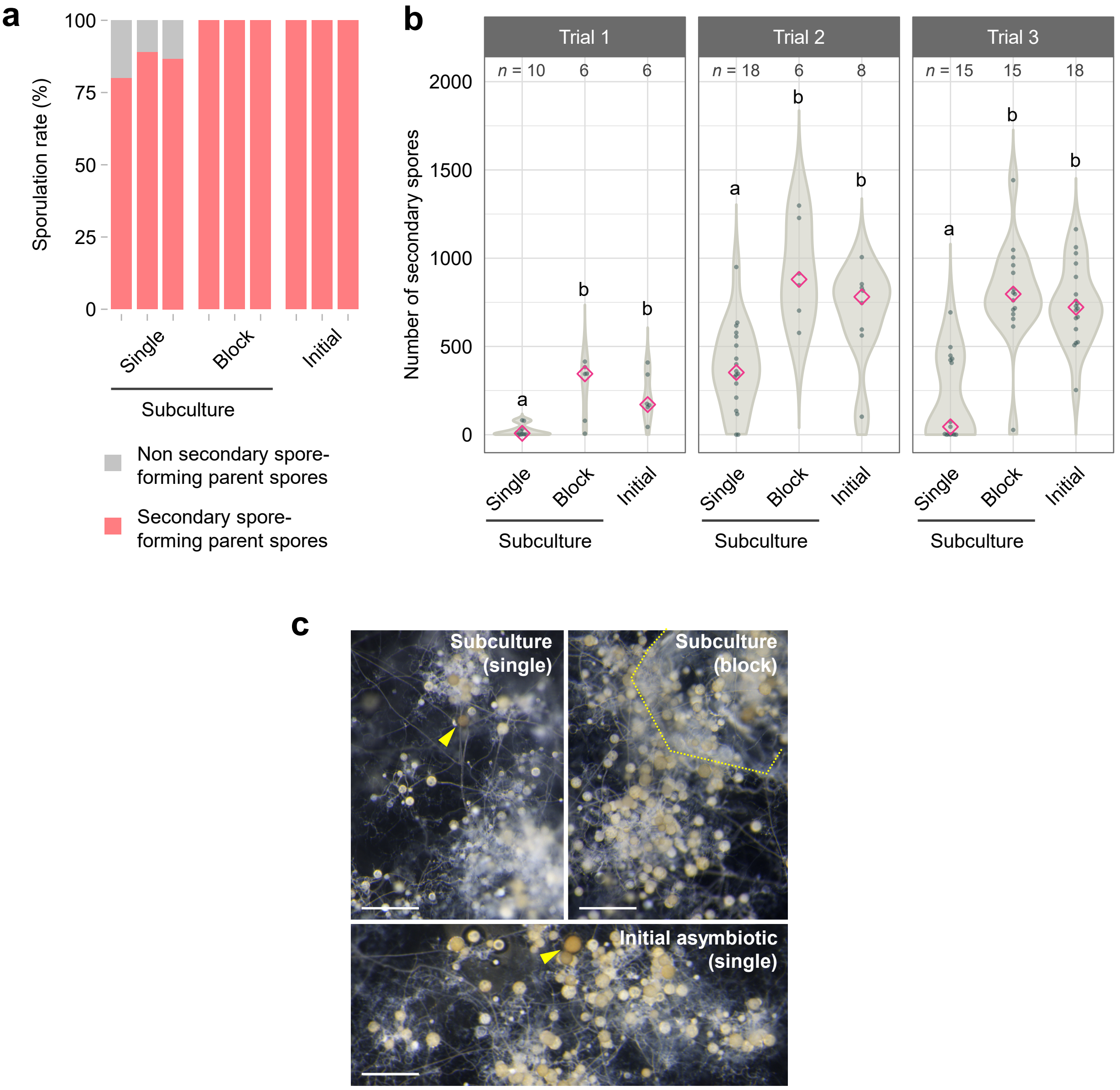

Supplementary Figure 9. Asymbiotic subculture of *R. clarus*. The comparison among secondary spore numbers in the initial asymbiotic culture and its subcultures. Single, a single asymbiotically-generated spore was placed on the medium. Block, about 5 mm square gel cut from the initial asymbiotic culture medium containing 20–40 spores was placed on the medium. Both initial and subcultures were performed on TGM medium. **a**, Sporulation rate, percentage of secondary spore-forming parent spores relative to germinated patent spores at 6 WAI. **b**, Numbers of secondary spores at 6 WAI. Some block inocula produced too many secondary spores for manual counting; only countable cases (with relatively low number secondary spores) were included. Diamonds indicate medians. Statistical significance was calculated using the Wilcoxon rank-sum test with Bonferroni correction. *Different letters* indicate significant differences ($p < 0.05$). p-values are described in Supplementary Data 2. **c**, Secondary spores in the initial asymbiotic culture and its subcultures from single parent spores (arrowheads) or gel block inoculum (dotted polygon) at 8 WAI. Bars = 500 µm.

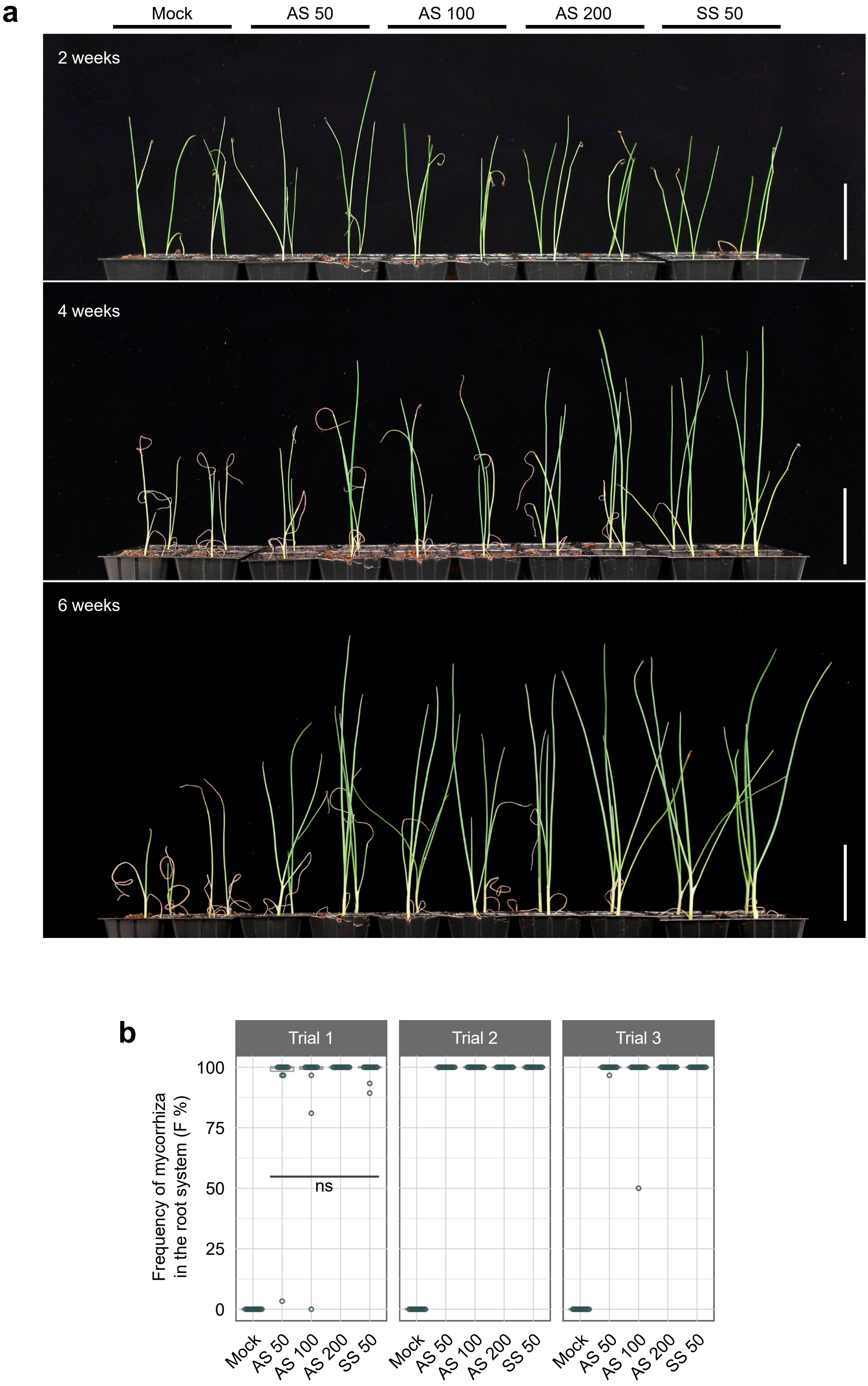
a
Mock
AS 50
AS 100
AS 200
SS 50
2 weeks
4 weeks
6 weeks
b
Trial 1
Trial 2
Trial 3
Frequency of mycorrhiza
in the root system (F %)
100
75
50
25
0
ns
Mock
AS 50
AS 100
AS 200
SS 50

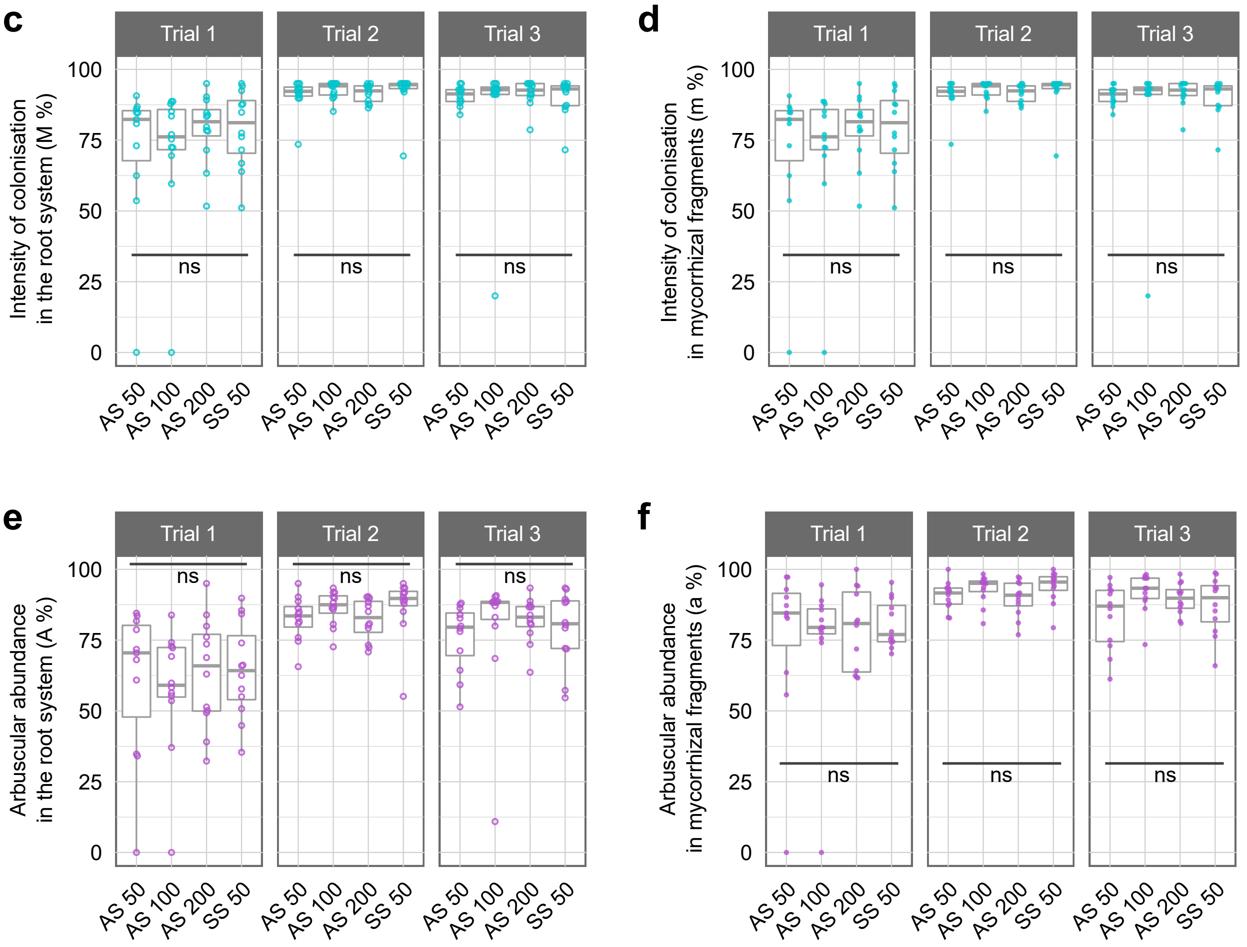

Supplementary Figure 10. Inoculation tests of *R. clarus*. a, Inoculation test to Welsh onions. The growth of plants from 2 to 6 weeks after inoculation. AS, asymbiotically-generated spores. SS, symbiotically-generated spores. Numbers following SS or AS are the number of spores for inoculation. Bars = 5 cm.**b–f**, Estimation of mycorrhizal colonization according to Trouvelet et al[47]. Roots sampled at 8 weeks after inoculation were analysed. The upper and lower side of boxes show the 25 and 75% quantiles, bars inside boxes indicate medians. Whiskers indicate 1.5 times the interquartile range. The statistical significance was calculated among all samples except mock treatments, using the Wilcoxon rank-sum test with Bonferroni correction ($p < 0.05$). ns, not significant.There were no significant differences among all conditions in all indicators. The number of tested root fragments and p-values are described in Supplementary Data 1 and 2, respectively.

Supplementary Table 1. Composition of the modified medium for the asymbiotic culture experiments of *R. clarus* HR1.

	Modified M medium[12]	The modified medium in this study
**Chemicals**	**Final concentration**	**Final concentration**
$MgSO_4 \cdot 7H_2O$	731 mg l^{-1}	731 mg l^{-1}
KNO_3	80 mg l^{-1}	80 mg l^{-1}
KCl	65 mg l^{-1}	65 mg l^{-1}
KH_2PO_4	4.8 mg l^{-1}	4.8 mg l^{-1}
$Ca(NO_3)_2 \cdot 4H_2O$	288 mg l^{-1}	288 mg l^{-1}
Fe(III)-EDTA	8 mg l^{-1}	8 mg l^{-1}
$MnCl_2 \cdot 4H_2O$	3 mg l^{-1}	3 mg l^{-1}
$ZnSO_4 \cdot 7H_2O$	1.3 mg l^{-1}	1.3 mg l^{-1}
H_3BO_3	1.5 mg l^{-1}	1.5 mg l^{-1}
$CuSO_4 \cdot 5H_2O$	0.065 mg l^{-1}	0.065 mg l^{-1}
$Na_2MoO_4 \cdot 2H_2O$	0.0012 mg l^{-1}	0.0012 mg l^{-1}
KI	0.75 mg l^{-1}	0.75 mg l^{-1}
MES (pH 6.5)	10 mM	10 mM
Sucrose	10 g l^{-1}	1 g l^{-1}
Glucose	−	1 g l^{-1}
Glycine	3 mg l^{-1}	3 mg l^{-1}
Pyridoxine-HCl	0.1 mg l^{-1}	0.1 mg l^{-1}
Nicotinic acid	0.5 mg l^{-1}	0.5 mg l^{-1}
myo-inositol	50 mg l^{-1}	50 mg l^{-1}
Thiamine-HCl	0.1 mg l^{-1}	10 mg l^{-1}
Gellan gum (GelGro)	3.5 g l^{-1}	−
Phytagel (Sigma-Aldrich)	−	3 g l^{-1}

Supplementary Table 2. Composition of the modified Long Ashton medium.
After mixing all compounds, pH was adjusted to 6.8 by adding NaOH.

Chemicals	Final concentration
$Ca(NO_3)_2 \cdot 4H_2O$	354 mg l^{-1}
KCl	54 mg l^{-1}
KH_2PO_4	2.7 mg l^{-1}
Fe(III)-EDTA	42 mg l^{-1}
$MgSO_4 \cdot 7H_2O$	185 mg l^{-1}
KI	1.2 mg l^{-1}
$MnCl_2 \cdot 4H_2O$	14 mg l^{-1}
H_3BO_3	22 mg l^{-1}
$ZnCl_2$	1.7 mg l^{-1}
$CuCl_2 \cdot 2H_2O$	0.47 mg l^{-1}
$CoCl_2 \cdot 6H_2O$	0.17 mg l^{-1}
$Na_2MoO_4 \cdot 2H_2O$	1.4 mg l^{-1}
KNO_3	101 mg l^{-1}
PIPES (pH7.5)	305 mg l^{-1}
