# Supplementary material for: Asymbiotic mass production of the arbuscular mycorrhizal fungus Rhizophagus clarus
Source: Commun Biol. 2022 Jan 12;5:43. doi: 10.1038/s42003-021-02967-5 (PMC8755765; doi:10.1038/s42003-021-02967-5)
Supplement: Supplementary file 3 — Description of Additional Supplementary Files [file 42003_2021_2967_MOESM3_ESM.pdf]

Description of Additional Supplementary Files

File name: Supplementary Movie 1

Description: A video of *R. clarus* HR1 developmental pattern, which was generated by images taken at 2 hour-intervals over time for 8 weeks by time lapse microscopy. The number at the bottom left indicates days after germ tube emergence.

File name: Supplementary Data 1

Description: Experimental conditions.

File name: Supplementary Data 2

Description: *p*-values and effect sizes.
